# Characteristics of Pediatric Emergency Department Visits in the Past 22 Years in the United States (1997–2019)

**DOI:** 10.7759/cureus.83310

**Published:** 2025-05-01

**Authors:** Zainab T O. Omar, Cynthia C Anyakorah, Okelue E Okobi, Kingsley C Okereke, Habeebah T Abubakar, Oscar O Ahumaraeze, Blessing O Oyewole

**Affiliations:** 1 Pediatrics, St. Joseph's Regional Medical Center, Paterson, USA; 2 Internal Medicine, Avalon University School of Medicine, Willemstad, UMI; 3 Family Medicine, IMG Research Academy and Consulting, Homestead, USA; 4 Family Medicine, Larkin Community Hospital Palm Springs Campus, Miami, USA; 5 Family Medicine, Lakeside Medical Center, Belle Glade, USA; 6 General Medicine, Enugu State University College of Medicine, Enugu, NGA; 7 General Practice, Ahfad University for Women, Omdurman, SDN; 8 Ophthalmology (Pediatrics), All Saints University School of Medicine, Roseau, DMA; 9 Family Medicine, Northfield Family Health, Canada, CAN

**Keywords:** emergency service, health survey, hospital utilization, pediatrics, united states

## Abstract

Background: Pediatric emergency department (ED) visits are an important indicator of children's health and healthcare access. Understanding the factors influencing these visits, including socioeconomic status, insurance coverage, and geographic location, is essential for improving pediatric care delivery.

Objective: This study aims to analyze the characteristics of pediatric ED visits in the United States over 22 years (1997-2019), focusing on factors such as poverty levels, health insurance status, geographic region, and frequency of ED visits.

Methods: A retrospective analysis of data from the National Health Interview Survey (NHIS) was conducted for children aged 0-17 years who had visited an ED at least once in the past 22 years. The study examined factors such as income as a percentage of the poverty level, health insurance status (insured, private, Medicaid, and uninsured), and geographic region (Northeast, Midwest, South, and West). Statistical analysis evaluated trends and associations between these characteristics and ED visit frequencies over time.

Results: From 1997 to 2019, the percentage of children visiting the ED fluctuated between 16.7% and 22.4%. The highest rate was observed in 2002 (22.4%) and the lowest in 2015 (16.9%). Children under six years had the highest ED visit rates, peaking in 2006 at 28.2%. Male children consistently had higher ED visit rates than females. Among racial groups, Black children had the highest rates, while Asian children had the lowest. Hispanic children had elevated ED visit rates, peaking in 2009. Children below the poverty level had higher visit rates, while those with Medicaid had consistently higher visits. Regional trends showed higher visits in the South, and urban areas reported more visits than rural ones. Multiple ED visits declined over the years, especially for children under six.

Conclusion: This study has disclosed that there is a significant relationship between pediatric ED visits and socioeconomic factors, insurance coverage, and geographic location. These findings emphasize the need for targeted interventions to address disparities in healthcare access and improve pediatric emergency care delivery across diverse populations.

## Introduction

The pediatric emergency department (ED) is an integral component of the healthcare system, providing essential care for acute and urgent medical conditions in children and adolescents. It manages a broad spectrum of presentations, from minor injuries to critical illnesses, underscoring its pivotal role in ensuring pediatric health outcomes [1-2]. Understanding the epidemiological and demographic patterns of pediatric ED utilization is crucial for optimizing healthcare delivery and resource allocation, particularly in addressing disparities in access to care, quality of services, and the evolving burden of disease [3].

In 2021, 140 million ED visits occurred in the United States. During that year, about 4% of children had two or more ED visits in the past 12 months, and 18% of adults had visited the ED in the past 12 months [4]. Epidemiologically, pediatric ED visits represent a significant portion of overall emergency care utilization. While many visits are for non-urgent conditions, a considerable proportion involve acute illnesses or injuries that require immediate medical attention [5]. Factors such as age, gender, socioeconomic status, insurance coverage, and geographic location contribute to the patterns observed in pediatric ED utilization [6]. Moreover, trends over time reveal shifts influenced by public health initiatives, changes in healthcare policies, and advancements in medical care. For instance, the introduction of vaccines and preventive measures has reduced the incidence of certain infectious diseases, while rising rates of pediatric mental health crises have led to increased ED visits for behavioral health concerns. These epidemiological patterns underscore the need for continual assessment of pediatric ED utilization to identify emerging healthcare needs and disparities [7].

The National Center for Health Statistics (NCHS) has been instrumental in gathering comprehensive data on ED utilization through initiatives like the National Hospital Ambulatory Medical Care Survey (NHAMCS). These datasets offer critical insights into patient demographics, visit reasons, diagnoses, and outcomes, enabling evidence-based decision-making to enhance healthcare delivery. Leveraging these data, researchers and policymakers can identify gaps in care, evaluate the impact of health interventions, and refine strategies to ensure equitable access to emergency services for the pediatric population [8].

This study examines the characteristics of pediatric ED visits in the United States from 1997 to 2019, focusing on demographic trends and clinical outcomes. Through this comprehensive analysis, the study seeks to inform targeted interventions and policy initiatives that improve pediatric ED utilization and address the challenges faced by this vulnerable population.

## Materials and methods

Data source and study design

This study utilized data from the NHAMCS, conducted by the NCHS. The NHAMCS is a nationally representative survey that collects data on visits to hospital EDs across the United States. A cross-sectional study design was employed to analyze pediatric ED visits over the 1997-2019 period, focusing on trends in patient demographics, visit characteristics, and outcomes.

Study participants and questionnaires

 The study data was drawn from the NHAMCS, which conducted national surveys to collect data on hospital ED visits. The data collected included children and adolescents aged 0-17 who visited an ED during the survey period. Data were collected through standardized questionnaires administered to hospital staff, capturing information on patient demographics, reasons for visits, diagnoses, treatments, and outcomes. Inclusion criteria required complete data on age, gender, and visit reasons, while records with significant missing information were excluded. Notably, the NHIS does not disclose exact annual sample sizes in the public-use datasets. However, the NHIS sampling methodology is designed to yield reliable national estimates based on stratified multistage sampling.

Data collection and quality assurance

Data were collected using a multistage probability sampling method to ensure national representativeness. Thus, multistage probability sampling entails stratification by demographics and geography, systematic sampling unit selection, and weighting adjustments to ensure the national representativeness and generalizability of the datasets. Quality assurance measures included rigorous training of data collectors, periodic validation checks, and standardized data entry protocols. The NCHS implemented systematic procedures to minimize errors and ensure data reliability, including double-checking records and cross-referencing with hospital logs.

Variables of interest

The primary focus of the study was on ED visits within the past 12 months among pediatrics aged under 18. Demographic data included age (categorized into three groups), gender (male/female), and race/ethnicity (White only, Black or African American only, American Indian or Alaska Native only, Asian only, Native Hawaiian or other Pacific Islander only), Hispanic origin and race (Hispanic or Latino, not Hispanic or Latino). Socioeconomic factors, such as poverty level (four categories: below 100%, 100-199%, 200-399%, and 400% or more), insurance status, geographic region (Northeast, Midwest, South, and West), location of residence such as metropolitan statistical areas (MSAs), and children with two or more emergency visits (categorized into three categories) were also analyzed. Notably, poverty level in the NHIS is determined by calculating the family’s total income as a percentage of the federal poverty threshold for a given calendar year, adjusted for family size and composition. The measure, known as the "poverty ratio," is then categorized into groups, such as poor (<100% of the federal poverty level), near-poor (100%-199%), and not poor (≥200%). These poverty classifications are used to examine disparities in ED visit patterns across socioeconomic strata.

Data analysis and statistical methods

Descriptive statistics were used to summarize the characteristics of pediatric ED visits. Trends over time were analyzed using weighted estimates to account for the survey’s complex sampling design. Chi-square tests and logistic regression analyses were conducted to assess associations between patient demographic variables (age, race/ethnicity, sex, and insurance status) and visit outcomes, including hospitalization and resource utilization. Furthermore, the results were expressed as adjusted odds ratios (aORs) with 95% confidence intervals, even as the statistical significance was set at p < 0.05. Data were analyzed using IBM SPSS Statistics for Windows (IBM Corp., Armonk, NY), ensuring accurate and efficient processing.

Ethical considerations

The study was conducted in compliance with ethical guidelines and regulations. The NHAMCS data used are publicly available and de-identified, ensuring patient confidentiality. Institutional review board (IRB) approval was not required for this secondary data analysis, as it involved no direct interaction with human subjects.

## Results

The analysis of pediatric ED visits over a 22-year period (1997-2019) revealed notable trends across various demographic and socioeconomic factors. These trends were observed by gender, race/ethnicity, poverty level, insurance status, and geographic region, with significant differences in ED utilization patterns based on these factors and reveled significant association between all variable over the years (p < 0.05).

The proportion of children under 18 years visiting the ED fluctuated between 1997 and 2019. It started at 19.9% ± 0.4 in 1997, peaked at 22.4% ± 0.5 in 2002, and gradually declined to 16.9% ± 0.5 in 2015, before slightly rising to 18.0% ± 0.5 in 2019. Children under six years consistently had higher ED visit rates compared to older age groups, with their proportion reaching a high of 28.2% ± 1.0 in 2006, then steadily declining to 22.7% ± 1.0 in 2019. By contrast, children aged six to 17 years began at 17.7% ± 0.5 in 1997, dropped to their lowest point at 13.8% ± 0.5 in 2013, and ended at 15.8% ± 0.6 in 2019. Table 1 presents data on the percentage of various demographic attributes related to ED visits for children, including socioeconomic status, health insurance status, geographic region, and location of residence, as well as the percentage of children with two or more ED visits from 1997 to 2019.

**Table 1:** Study characteristics ED visits within the time period of 1997-2019 -: not available, ED: emergency department, MSA: metropolitan statistical area. A chi-square test was used in the calculation of the P-value.

Characteristic	1997	1998	1999	2000	2001	2002	2003	2004	2005	2006	2007	2008	2009	2010	2011	2012	2013	2014	2015	2016	2017	2018	2019	P value
Percent of children with one or more emergency department visits: overall																								
Under 18 years	19.9±0.4	20.2±0.4	17.9±0.4	20.3±0.4	20.6±0.4	22.4±0.5	20.9±0.5	20.9±0.5	20.5±0.5	21.3±0.6	20.2±0.5	20.9±0.6	20.8±0.5	22.1±0.5	18.5±0.4	17.8±0.4	17.6±0.4	16.7±0.5	16.9±0.5	17.5±0.5	16.9±0.5	19.6±0.5	18.0±0.5	<0.05
Under six years	24.3±0.7	25.2±0.8	23.3±0.7	25.7±0.8	25.0±0.8	28.0±0.8	26.5±0.8	26.2±0.8	26.8±0.9	28.2±1.0	23.9±1.0	27.4±1.0	25.9±0.9	27.8±0.8	24.2±0.8	24.4±0.8	23.6±0.8	22.6±0.8	21.7±0.9	22.0±0.9	22.2±1.0	23.9±1.0	22.7±1.0	
Six to 17 years	17.7±0.5	17.8±0.5	15.3±0.5	17.6±0.5	18.6±0.5	19.7±0.5	18.2±0.5	18.4±0.5	17.4±0.5	17.9±0.7	18.3±0.6	17.5±0.6	18.2±0.6	19.1±0.6	15.6±0.5	14.6±0.5	14.8±0.5	13.8±0.5	14.5±0.5	15.3±0.6	14.4±0.5	17.6±0.7	15.8±0.6	
Percent of children with one or more emergency department visits: based on sex																								
Male	21.5 ± 0.6	22.1 ± 0.6	19 ± 0.6	21.5 ± 0.6	21.9 ± 0.6	23.7 ± 0.7	22.4 ± 0.6	21.9 ± 0.6	21.7 ± 0.6	22.5 ± 0.9	21.6 ± 0.7	21.9 ± 0.8	22.2 ± 0.8	23.3 ± 0.7	18.9 ± 0.6	18.4 ± 0.6	18.4 ± 0.6	16.9 ± 0.6	17.3 ± 0.6	18.7 ± 0.7	17.2 ± 0.7	21.1 ± 0.8	17.9 ± 0.7	<0.05
Female	18.3 ± 0.6	18.3 ± 0.6	16.8 ± 0.6	19 ± 0.6	19.3 ± 0.6	21.1 ± 0.6	19.3 ± 0.6	20 ± 0.6	19.2 ± 0.6	20 ± 0.8	18.8 ± 0.7	19.7 ± 0.8	19.4 ± 0.7	20.9 ± 0.7	18.1 ± 0.6	17.2 ± 0.6	16.9 ± 0.6	16.4 ± 0.6	16.3 ± 0.6	16.2 ± 0.7	16.6 ± 0.7	18.1 ± 0.7	18.2 ± 0.7	
Percent of children with one or more emergency department visits: based on race																								
White only	19.4 ± 0.5	19.8 ± 0.5	17.1 ± 0.5	19.9 ± 0.5	20.1 ± 0.5	21.3 ± 0.5	20.3 ± 0.5	20.6 ± 0.5	19.8 ± 0.5	21.2 ± 0.7	20 ± 0.6	20.2 ± 0.6	19.8 ± 0.6	21.2 ± 0.6	17.9 ± 0.5	16.8 ± 0.5	17.1 ± 0.5	15.9 ± 0.5	16.3 ± 0.5	16.6 ± 0.5	16.1 ± 0.5	18.4 ± 0.6	17 ± 0.6	<0.05
Black or African American only	24 ± 1.1	24.1 ± 1.1	22.5 ± 1.1	22.7 ± 1.1	22.7 ± 1	27.9 ± 1.2	23.9 ± 1.1	23.1 ± 1.2	23.8 ± 1.1	25 ± 1.3	23.1 ± 1.3	24.2 ± 1.4	26.9 ± 1.3	27.6 ± 1.3	22.6 ± 1.2	24.1 ± 1.2	22.6 ± 1.2	21.3 ± 1.2	21.6 ± 1.3	21.8 ± 1.4	20 ± 1.4	26.3 ± 1.6	22.9 ± 1.7	
American Indian or Alaska Native only	24.1 ± 5.8	29 ± 4.8	33.3 ± 6.1	38 ± 7	26.9 ± 5.1	-	22.7 ± 5.5	17.7 ± 3.6	32.1 ± 7.3	19.7 ± 5.4	22 ± 3.9	35.6 ± 6	23.1 ± 4.9	20.9 ± 3.8	21.9 ± 3.4	22.4 ± 5.4	18.8 ± 3.3	28.7 ± 4.5	23.3 ± 3.5	34.4 ± 5.5	27.6 ± 5.2	35.6 ± 5.5	16.8 ± 4.1	
Asian only	-	-	9.4 ± 1.6	12.3 ± 1.9	11.4 ± 1.8	14.4 ± 2.1	14.2 ± 2.3	15.8 ± 2.1	14.6 ± 1.9	13.4 ± 1.7	11.4 ± 1.7	12 ± 1.6	11.4 ± 1.5	15 ± 1.7	9.3 ± 1.2	8.8 ± 1.2	8.8 ± 1.2	10.4 ± 1.2	9.2 ± 1.2	8.4 ± 1.3	10.1 ± 1.6	12.2 ± 1.8	10.2 ± 1.4	
Percent of children with one or more emergency department visits: based on Hispanic origin																								
Hispanic or Latino	21.1 ± 0.8	19 ± 0.8	15.9 ± 0.8	18.6 ± 0.8	19.3 ± 0.8	20.6 ± 0.9	20.3 ± 0.9	20.6 ± 0.9	19.5 ± 0.8	19.7 ± 1	18 ± 0.9	21.6 ± 1.1	20.2 ± 1	23.6 ± 0.9	19.2 ± 0.8	16.8 ± 0.7	17.3 ± 0.8	17.1 ± 0.8	18.8 ± 0.9	17.8 ± 1	18.2 ± 1.1	20.7 ± 1.2	20.5 ± 1.1	<0.05
Not Hispanic or Latino	19.7 ± 0.5	20.5 ± 0.5	18.3 ± 0.5	20.6 ± 0.5	20.9 ± 0.5	22.8 ± 0.5	21 ± 0.5	21 ± 0.5	20.7 ± 0.5	21.7 ± 0.7	20.8 ± 0.7	20.7 ± 0.6	21 ± 0.6	21.7 ± 0.6	18.3 ± 0.5	18.1 ± 0.5	17.8 ± 0.5	16.6 ± 0.5	16.2 ± 0.5	17.4 ± 0.5	16.5 ± 0.5	19.2 ± 0.6	17.2 ± 0.6	
Percent of children with one or more emergency department visits: based on poverty level																								
Below 100%	25.1 ± 1.0	26.6 ± 1.1	23.5 ± 1.1	25 ± 1.1	24.9 ± 1.1	26.7 ± 1.2	26.9 ± 1.2	27.6 ± 1.3	27.3 ± 1.2	25.8 ± 1.4	28.1 ± 1.5	27.7 ± 1.5	26.6 ± 1.3	30.6 ± 1.3	24.9 ± 1.0	24.9 ± 1.1	24.1 ± 1.1	24.7 ± 1.2	23.1 ± 1.1	25.1 ± 1.3	24.1 ± 1.3	26 ± 1.6	25.3 ± 1.5	<0.05
100%–199%	22 ± 0.9	22.7 ± 1.0	21.3 ± 1.0	22.4 ± 1.0	21.9 ± 0.9	26.6 ± 1.1	22.8 ± 1.0	22.3 ± 0.9	21.8 ± 1.0	22.1 ± 1.2	22.5 ± 1.2	22.2 ± 1.2	23.3 ± 1.1	25.7 ± 1.1	19.8 ± 0.9	19.7 ± 0.9	20.1 ± 1.0	18.5 ± 0.9	18.7 ± 1.0	20.3 ± 1.1	20.7 ± 1.2	23.7 ± 1.4	22.9 ± 1.2	
200%–399%	18 ± 0.7	17.5 ± 0.7	15.7 ± 0.7	19 ± 0.7	20.8 ± 0.8	19.8 ± 0.8	19.9 ± 0.8	18.8 ± 0.8	18.9 ± 0.8	20.4 ± 1.1	17.8 ± 0.9	20.1 ± 1.0	18.9 ± 1.0	18.4 ± 0.9	15.9 ± 0.7	15 ± 0.8	15.5 ± 0.8	13.8 ± 0.9	14.7 ± 0.8	15.2 ± 0.8	15.3 ± 0.8	18.5 ± 1.0	15.1 ± 0.8	
400% or more	16.3 ± 0.7	17.1 ± 0.7	14.3 ± 0.7	17 ± 0.7	16.9 ± 0.7	19.4 ± 0.8	16.7 ± 0.7	18 ± 0.8	16.8 ± 0.7	18.1 ± 1.0	15.5 ± 0.9	16.1 ± 0.9	16 ± 1.0	15.9 ± 0.8	14.6 ± 0.7	12.9 ± 0.8	12.3 ± 0.7	11.4 ± 0.7	12.9 ± 0.8	12.5 ± 0.8	11.4 ± 0.7	14 ± 0.8	12.9 ± 0.7	
Percent of children with one or more emergency department visits: based on the health insurance status																								
Insured	19.8 ± 0.4	20.2 ± 0.5	18.1 ± 0.4	20.7 ± 0.5	20.9 ± 0.5	22.9 ± 0.5	21.4 ± 0.5	21 ± 0.5	20.7 ± 0.5	21.9 ± 0.6	20.5 ± 0.6	21.2 ± 0.6	21.1 ± 0.5	22.3 ± 0.5	18.8 ± 0.5	18 ± 0.5	17.8 ± 0.5	16.9 ± 0.5	16.9 ± 0.5	17.5 ± 0.5	17.1 ± 0.5	19.7 ± 0.6	18.2 ± 0.5	<0.05
Private	17.5 ± 0.5	17.9 ± 0.5	15.4 ± 0.5	18.4 ± 0.5	18.6 ± 0.5	20 ± 0.5	18.1 ± 0.5	18.5 ± 0.5	17.4 ± 0.5	19.2 ± 0.8	17.1 ± 0.6	16.8 ± 0.6	16.6 ± 0.6	17.1 ± 0.6	14.9 ± 0.5	13 ± 0.6	13.2 ± 0.5	12.4 ± 0.6	12.5 ± 0.6	13.2 ± 0.5	12.6 ± 0.6	15.6 ± 0.6	13.5 ± 0.6	
Medicaid	28.2 ± 1.1	29 ± 1.2	28.8 ± 1.2	28.6 ± 1.1	28.3 ± 1.1	30.7 ± 1.0	28.9 ± 1.0	27.2 ± 1.0	28.5 ± 1.0	27.2 ± 1.1	27.3 ± 1.1	29.1 ± 1.1	27.8 ± 1.0	30 ± 1.0	24.4 ± 0.8	24.8 ± 0.8	24 ± 0.8	22.9 ± 0.8	22.8 ± 0.8	23.4 ± 0.9	23.3 ± 0.9	26.1 ± 1.0	25 ± 1.0	
Uninsured	20.2 ± 1.0	20.1 ± 1.1	16.4 ± 1.1	17.2 ± 1.1	17.3 ± 1.2	18.2 ± 1.3	17.1 ± 1.2	20.7 ± 1.6	18.4 ± 1.4	16.8 ± 1.4	17.7 ± 1.6	18.4 ± 1.8	16.8 ± 1.6	19.4 ± 1.6	13.8 ± 1.3	15.6 ± 1.5	15.1 ± 1.6	14.7 ± 1.7	14.3 ± 1.8	16.6 ± 2.4	14.1 ± 2.0	18.1 ± 2.3	15.4 ± 2.1	
Percent of children with one or more emergency department visits: based on geographic region																								
Northeast	18.5 ± 0.9	18.5 ± 0.9	17.1 ± 1.0	19.7 ± 0.9	20.2 ± 1.0	23.3 ± 1.1	21.8 ± 1.0	24.2 ± 1.2	20.9 ± 1.0	24.1 ± 1.3	22.3 ± 1.2	21.3 ± 1.5	21.9 ± 1.3	22.3 ± 1.2	19.1 ± 1.2	17.1 ± 1.1	19.3 ± 1.2	17.6 ± 1.2	16.5 ± 1.3	17.5 ± 1.3	14.7 ± 1.0	19 ± 1.3	20.2 ± 1.4	<0.05
Midwest	19.5 ± 0.9	20.1 ± 0.9	18.4 ± 0.9	20.3 ± 0.9	21.2 ± 0.9	22.7 ± 0.9	21.4 ± 1.0	21 ± 0.9	21.8 ± 1.0	22.4 ± 1.4	21.2 ± 1.3	22 ± 1.2	22 ± 1.0	23.3 ± 1.2	19 ± 1.0	17.9 ± 1.0	18.5 ± 1.0	18.7 ± 1.2	19.1 ± 1.0	20.2 ± 1.1	18.4 ± 1.1	19.8 ± 1.1	17.3 ± 1.1	
South	21.8 ± 0.7	22.9 ± 0.8	19.2 ± 0.7	21.9 ± 0.7	22.2 ± 0.7	24.4 ± 0.8	21.7 ± 0.8	21.5 ± 0.8	21.7 ± 0.8	22.8 ± 1.0	21.4 ± 0.9	21.5 ± 0.9	22.3 ± 0.9	23.4 ± 0.9	19.8 ± 0.8	19.6 ± 0.8	18.2 ± 0.7	16.7 ± 0.7	17.6 ± 0.8	16.4 ± 0.7	17.7 ± 0.8	21.3 ± 0.8	19 ± 0.9	
West	18.5 ± 0.9	17.6 ± 0.8	15.9 ± 0.8	18 ± 0.8	17.9 ± 0.9	17.9 ± 0.8	18.3 ± 0.8	17.6 ± 0.8	16.7 ± 0.9	15.4 ± 0.9	15.7 ± 0.9	18.6 ± 1.1	16.6 ± 0.9	19.1 ± 0.9	15.7 ± 0.7	15.5 ± 0.8	14.8 ± 0.8	14.2 ± 0.8	13.7 ± 0.7	16.7 ± 0.9	16 ± 1.0	17.3 ± 1.1	15.7 ± 1.0	
Percent of children with one or more emergency department visits: based on the location of residence																								
Within MSA	19.7 ± 0.4	19.6 ± 0.5	16.7 ± 0.4	19.9 ± 0.5	19.8 ± 0.5	21.7 ± 0.5	20.2 ± 0.5	20.5 ± 0.5	20 ± 0.5	20.8 ± 0.6	19.6 ± 0.6	19.9 ± 0.6	20.2 ± 0.6	21.8 ± 0.6	17.9 ± 0.5	17.5 ± 0.5	17 ± 0.5	16.5 ± 0.5	16.4 ± 0.5	16.7 ± 0.5	16.3 ± 0.5	19.2 ± 0.6	17.8 ± 0.6	<0.05
Outside MSA	20.8 ± 1.0	22.7 ± 1.1	22.4 ± 1.0	21.9 ± 1.0	24.2 ± 1.2	25.4 ± 1.1	23.6 ± 1.1	23 ± 1.1	22.4 ± 1.3	23.9 ± 1.7	23.4 ± 1.5	25.7 ± 1.6	24.2 ± 1.3	24.2 ± 1.4	21.9 ± 1.1	19.7 ± 1.0	21.2 ± 1.4	17.8 ± 1.3	20 ± 1.4	22.2 ± 1.5	21 ± 1.2	22.9 ± 1.7	19.4 ± 1.3	

Based on gender

**Figure 1:**
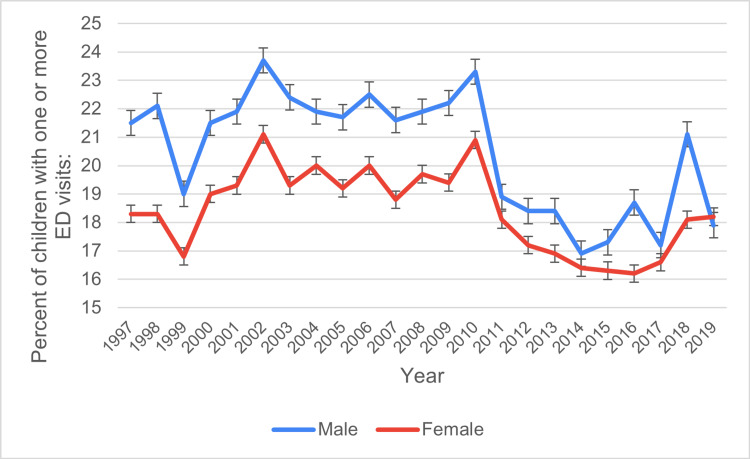
ED visit trends based on gender during the study period ED: emergency department

From 1997 to 2019, males consistently exhibited higher ED visit percentages than females. Male values began at 21.5% ± 0.6 in 1997, peaked at 23.7% ± 0.7 in 2002, and declined steadily to 17.9% ± 0.7 by 2019. Similarly, females started at 18.3% ± 0.6 in 1997, reached a high of 21.1% ± 0.6 in 2002, and decreased gradually to 18.2% ± 0.7 by 2019. Both genders demonstrated a similar trend, with peaks in 2002 followed by steady declines, although the decrease was more pronounced among males, reflecting persistent but narrowing gender disparities in ED visit percentages over time. Figure 1 shows the ED visit trends based on gender during study period.

Based on race

**Figure 2:**
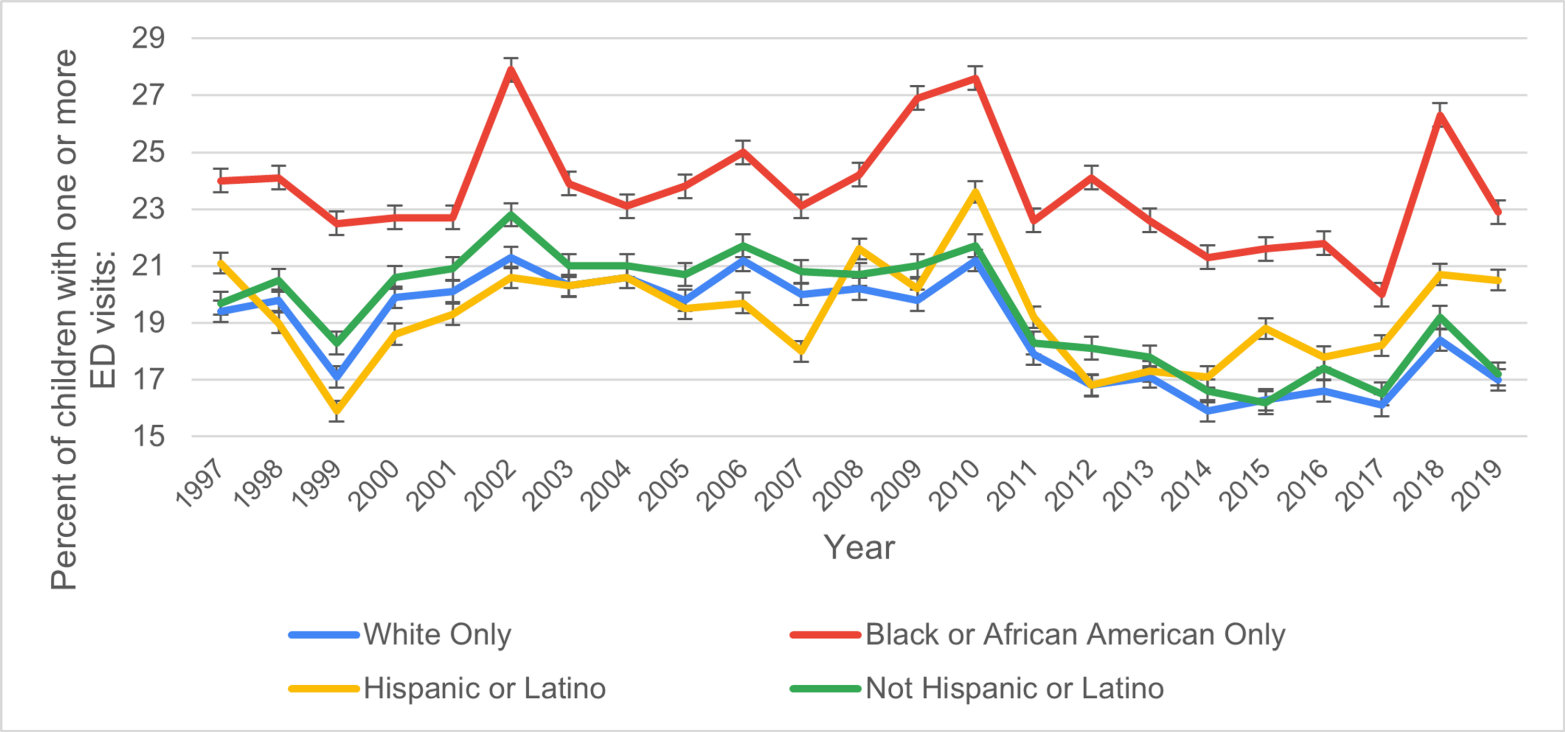
ED visit trends based on race during the study period ED: emergency department

Racial disparities in ED visit rates were evident throughout the study period. Black or African American children consistently recorded the highest rates, starting at 24% ± 1.1 in 1997, peaking at 27.9% ± 1.2 in 2002, and declining to 22.9% ± 1.7 by 2019. White children exhibited a moderate decline, with rates decreasing from 19.4% ± 0.5 in 1997 to 17% ± 0.6 in 2019, punctuated by minor fluctuations. Asian children had the lowest ED visit rates, beginning at 9.4% ± 1.6 in 1999, peaking at 15.8% ± 2.1 in 2004, and ending at 10.2% ± 1.4 in 2019. These trends highlight persistent disparities among racial groups, with Black and American Indian or Alaska Native children consistently exhibiting higher rates than other racial groups. Figure 2 shows the ED visit trends based on race during study period.

Based on Hispanic or Latino and race

Hispanic or Latino children showed a distinct pattern, beginning with a rate of 21.1% ± 0.8 in 1997, peaking at 23.6% ± 0.9 in 2010, and stabilizing at 20.5% ± 1.1 in 2019 (Table 1). Non-Hispanic children followed a similar downward trajectory, starting at 19.7% ± 0.5 in 1997, peaking at 22.8% ± 0.5 in 2002, and declining to 17.2% ± 0.6 by 2019. Overall, Hispanic or Latino children consistently exhibited higher ED visit rates than their non-Hispanic counterparts. These findings underscore the nuanced patterns in ED utilization among racial and ethnic groups, with notable disparities persisting across demographic subgroups over time.

Based on the poverty level

**Figure 3:**
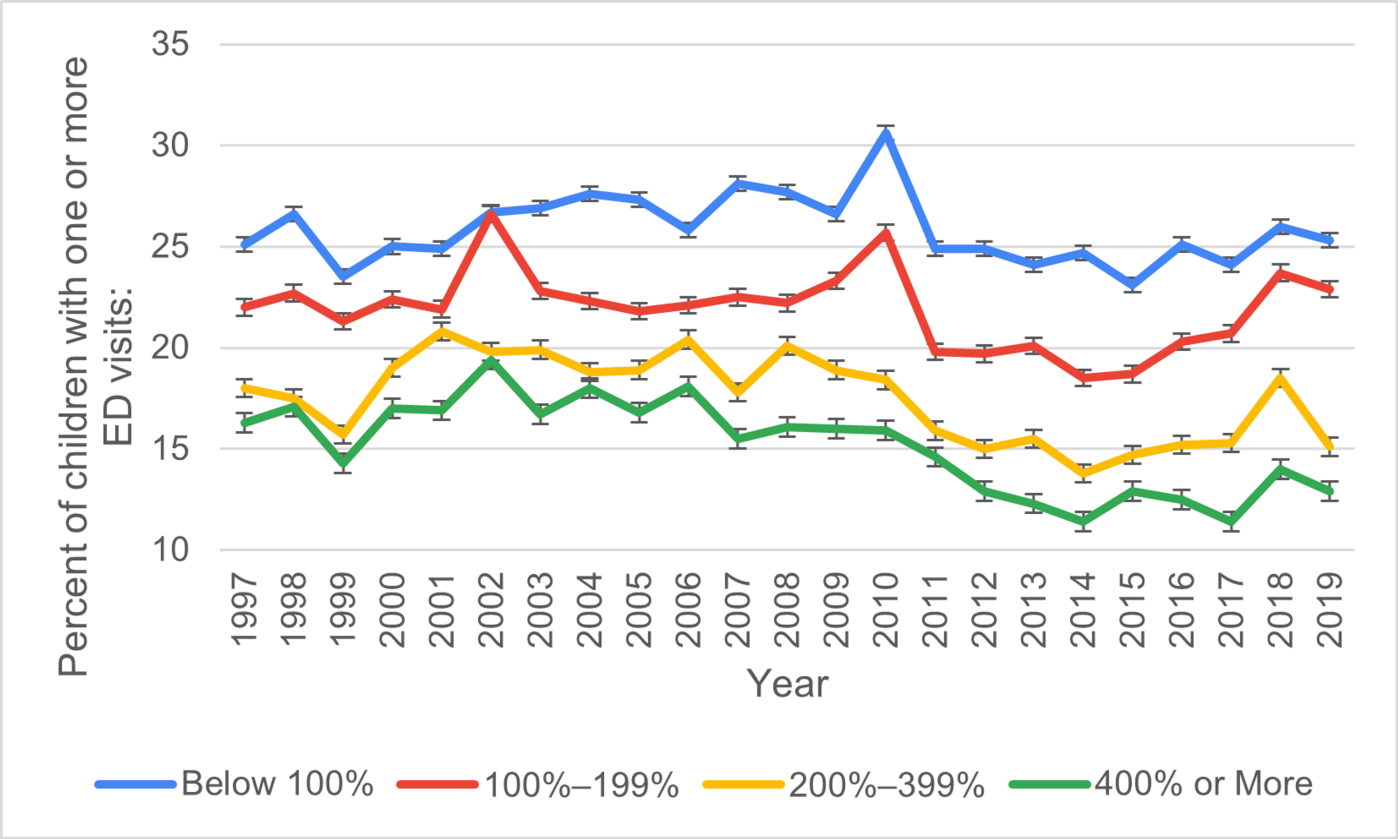
ED visit trends based on poverty level during the study period ED: emergency department

The percentage of pediatric ED visits among children categorized as poor, with family incomes of <100% FPL fluctuated over the years, peaking in 2010 at 30.6 ± 1.3% and gradually declining to 25.3 ± 1.5% by 2019. For children categorized as low income, between 100% and 199% FPL, the percentages remained comparatively stable, declining slightly from 22 ± 0.9% in 1997 to 22.9 ± 1.2% in 2019. Children categorized as middle income, between 200% and 399% FPL, a substantial decrement was reported, starting from 18 ± 0.7% in 1997, and peaking at 20.8 ± 0.8% in 2001, and further reducing to 15.1 ± 0.8% by 2019. Finally, children categorized as high income, ≥400% FPL, indicated a significant decline in ED visits from 16.3 ± 0.7% in 1997 to 12.9 ± 0.7% by 2019, reflecting an overall downward trends over the years. Figure 3 below illustrates the ED visit trends based on federal poverty level during the study period.

Based on health insurance status at the time of interview

**Figure 4:**
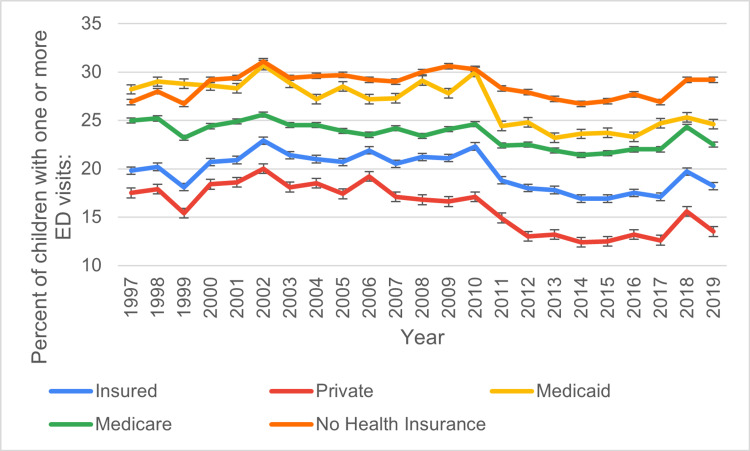
ED visit trends based on the health insurance status at the time of interview during the study period ED: emergency department

Pediatric ED visits among insured children peaked at 22.9 ± 0.5% in 2002 but declined steadily to 18.2 ± 0.5% by 2019. Visits among privately insured children showed a similar trend, decreasing from 17.5 ± 0.5% in 1997 to 13.5 ± 0.6% by 2019. Medicaid-covered children consistently reported higher visit percentages, peaking at 30.7 ± 1.0% in 2002 and ending at 25 ± 1.0% in 2019. Similar trends were observed in Medicare-insured patients, peaking at 24.7 ± 1.0% in 2002 and ending at 23.2 ± 1.0% in 2019. Uninsured children showed fluctuations, starting at 20.2 ± 1.0% in 1997, dropping to 14.1 ± 2.0% in 2017, and ending at 15.4 ± 2.1% in 2019, highlighting an overall decrease. Figure 4 shows the ED visit trends based on the health insurance status at the time of interview during the study period.

Based on the geographic region

The South consistently recorded the highest ED visits, peaking at 24.4 ± 0.8% in 2002 and ending at 19 ± 0.9% in 2019 (Table 1). The Midwest and Northeast showed similar trends, with the Midwest starting at 19.5 ± 0.9% in 1997 and declining to 17.3 ± 1.1% by 2019. The Northeast saw a peak of 24.2 ± 1.2% in 2004 and dropped to 20.2 ± 1.4% by 2019. The West exhibited the lowest percentages, decreasing from 18.5 ± 0.9% in 1997 to 15.7 ± 1.0% by 2019.

Based on the location of residence

Children residing within MSA showed a consistent decline in ED visits, starting at 19.7 ± 0.4% in 1997 and ending at 17.8 ± 0.6% in 2019 (Table 1). By contrast, children outside MSAs reported higher percentages, with peaks at 25.7 ± 1.6% in 2008 and a gradual decline to 19.4 ± 1.3% by 2019. This disparity highlights the influence of urban and rural residence on healthcare access.

## Discussion

This study analyzes trends in pediatric ED visits from 1997 to 2019, highlighting the role of sociodemographic factors, including age, sex, race, poverty level, health insurance status, and geographic region. Over the years, the percentage of children under 18 years making at least one ED visit fluctuated, revealing important insights about healthcare access and utilization in pediatric populations.

The age breakdown of ED visits reveals distinct patterns, particularly when comparing younger children (under six years) to older children (six to 17 years). In 1997, 24.3% of children under six years visited the ED, a number that rose to 28.0% in 2002. However, by 2019, this figure decreased to 22.7%. This trend of initially increasing visits followed by a decline in more recent years may be associated with various factors, including improvements in preventive healthcare measures for young children, greater access to outpatient care, and shifts in healthcare policies favoring early intervention and community-based healthcare [9].

Consequently, the percentage of ED visits among children aged six to 17 years exhibited a more stable trend, although there was a gradual decrease from 17.7% in 1997 to 15.8% in 2019. This age group may be more likely to access healthcare through other channels, such as urgent care centers, family physicians, or school-based health services, leading to fewer ED visits over time. Furthermore, as healthcare systems evolve and more services become available in non-hospital settings, children in this age group may increasingly turn to these options for minor injuries or illnesses rather than EDs [10-11].

The gender analysis revealed that male children consistently exhibited higher rates of ED visits compared to female children. In 1997, 21.5% of male children visited the ED, compared to 18.3% of female children. The gap between male and female ED visits remained throughout the study period, although both genders showed a downward trend in ED visits over time. By 2019, 17.9% of male children visited the ED, while 18.2% of female children did. This consistent difference between genders is likely related to behavioral factors, as boys tend to engage in riskier physical activities, leading to a higher incidence of injuries that may require emergency care. Studies such as Alpern et al. have shown that male children generally experience higher rates of trauma and injury, which are common reasons for ED visits [12-14].

Race and ethnicity also played a significant role in pediatric ED utilization. White children consistently had the lowest percentage of ED visits compared to Black, Hispanic, and American Indian children. For instance, in 1997, 19.4% of White children visited the ED, while 24% of Black children did, and 21.1% of Hispanic children. These disparities reflect long-standing healthcare access inequalities, particularly among minority groups [10,12]. Black and Hispanic children are more likely to be from lower-income households, which can limit their access to primary care and lead to higher reliance on emergency services [15]. American Indian children, whose data were available sporadically in the dataset, showed even higher rates of ED visits, which may be linked to historical and systemic barriers in healthcare access, including limited access to facilities and culturally relevant healthcare [16].

Interestingly, Asian children, whose data was available from 1999 onward, showed a much lower percentage of ED visits compared to other racial groups, with a steady decline from 12.3% in 2000 to 10.2% in 2019. This could indicate differences in health-seeking behaviors, cultural attitudes toward healthcare, or better access to primary care among Asian populations. Previous studies like Ye et al. suggested that immigrant and refugee populations, often prevalent in Asian communities, may rely less on emergency services and more on traditional or community-based healthcare practices [17-18].

The analysis of the poverty level revealed a clear link between income and ED visit rates. Children from families living below 100% of the Federal Poverty Level (FPL) consistently had the highest ED visit rates. In 1997, 25.1% of children in this group visited the ED, which rose to 30.6% in 2013 before declining to 25.3% in 2019. Similarly, children from families earning 100% to 199% of the FPL showed higher ED utilization rates than those from higher-income families. These findings underscore the importance of socioeconomic status in determining access to healthcare. Families in lower-income brackets often face barriers to accessing routine healthcare, leading to greater reliance on emergency services for non-urgent conditions [19]. In contrast, children from families with higher incomes exhibited lower ED visit rates. For instance, only 18.9% of children from families with incomes greater than 400% of the FPL visited the ED in 1997, compared to 15.2% in 2019. This pattern suggests that wealthier families are better able to access primary care and preventive services, which may reduce the need for emergency care [20].

Insurance status was a significant factor in pediatric ED visits. Children with private insurance consistently had the lowest rates of ED utilization. In 1997, 17.5% of privately insured children visited the ED; by 2019, this number had dropped to 13.5%. This decline in ED visits among privately insured children may reflect the increased availability of primary care and preventive services and the use of urgent care centers or telemedicine services as alternatives to EDs [21].

Conversely, children covered by Medicaid showed the highest rates of ED visits. In 1997, 28.2% of Medicaid-enrolled children visited the ED, and while this number declined to 25.0% by 2019, it remained higher than the rates for children with private insurance [22]. Medicaid-enrolled children often come from lower-income households, where access to primary care is more limited. This lack of access can lead to increased reliance on emergency services, which are often used as a last resort for healthcare needs [23].

Uninsured children exhibited fluctuating patterns in ED utilization, with a decline in the percentage visiting the ED over time. In 1997, 20.2% of uninsured children visited the ED, but by 2019, this number had decreased to 15.4%. This decrease is likely attributed to the expansion of health insurance coverage, particularly through the Affordable Care Act (ACA), which aimed to reduce the number of uninsured children [24-25].

Geographic location also played a role in pediatric ED visit patterns. Children in the Northeast and Midwest exhibited higher ED visit rates than those in the South and West. In 1997, 18.5% of children in the Northeast and 19.5% in the Midwest visited the ED, compared to 17.8% in the South. By 2019, these trends persisted, with slight increases in ED visits in the Northeast. These regional differences could be attributed to variations in healthcare infrastructure, access to primary care, and population density [26]. Glanz et al. reported that under-vaccinated children had fewer outpatient visits in comparison to children vaccinated based on the recommended schedules (incidence rate ratio (IRR): 0.89; 95% CI: 0.89-0.90) [27]. Regardless of this, under-vaccinated children were observed to experience higher rates of inpatient hospitalizations compared to their age-appropriately vaccinated counterparts (IRR: 1.21; 95% CI: 1.18-1.23). Notably, among those under-vaccinated due to parental choice, both outpatient visits (IRR: 0.94; 95% CI: 0.93-0.95) and ED visits (IRR: 0.91; 95% CI: 0.88-0.94) were lower than in fully vaccinated children [27]. 

The findings highlight the critical need for strategies that address disparities in pediatric ED utilization. Future efforts should prioritize enhancing access to primary care and preventive services for socioeconomically disadvantaged and minority populations. Incorporating health education, improving Medicaid coverage, and streamlining care coordination are essential to mitigating overreliance on ED services. Moreover, it is crucial to investigate the role of social determinants of health and their impact on pediatric healthcare patterns. Physicians and healthcare policymakers must collaborate to develop targeted, evidence-based interventions aimed at reducing health disparities and optimizing resource allocation in emergency care for pediatric populations.

Strengths and limitations of this study

This study has several strengths. For instance, it leverages over two decades of nationally representative data from the NHIS, offering a comprehensive view of pediatric ED visit trends in the US. The use of standardized survey instruments and rigorous quality assurance protocols enhances the reliability of findings. In addition, the inclusion of multiple sociodemographic and geographic variables allows for a nuanced understanding of disparities in ED utilization. Analytical methods were applied using SPSS with appropriate weighting, ensuring accurate national estimates.

However, there are limitations. The NHIS is based on self-reported data from household interviews, which may be subject to recall bias or misreporting. The exact annual sample size is not disclosed in the public-use dataset, limiting the ability to assess precision at a granular level. Furthermore, the cross-sectional design precludes causal inference, and unmeasured confounding factors may influence associations observed between sociodemographic factors and ED visits.

## Conclusions

This study highlights key trends in pediatric ED visits from 1997 to 2019, revealing significant disparities across gender, race, socioeconomic status, insurance type, and geographic location. Male children, those from low-income families, and racial or ethnic minorities, including Black, Hispanic, and American Indian children, consistently exhibited higher ED utilization. Medicaid-insured and uninsured children also relied more on ED services compared to those with private insurance. These findings emphasize the need for targeted interventions to reduce disparities, improve access to primary care, and expand insurance coverage. Addressing these issues is essential to decreasing unnecessary ED visits and ensuring equitable healthcare access for all children, regardless of their socioeconomic or demographic background.
